# Physiological and Transcriptomic Analyses Revealed the Implications of Abscisic Acid in Mediating the Rate-Limiting Step for Photosynthetic Carbon Dioxide Utilisation in Response to Vapour Pressure Deficit in *Solanum Lycopersicum* (Tomato)

**DOI:** 10.3389/fpls.2021.745110

**Published:** 2021-11-10

**Authors:** Dalong Zhang, Qingjie Du, Po Sun, Jie Lou, Xiaotian Li, Qingming Li, Min Wei

**Affiliations:** ^1^College of Horticultural Science and Engineering, Shandong Agricultural University, Tai'an, China; ^2^State Key Laboratory of Crop Biology, Tai'an, China; ^3^Scientific Observing and Experimental Station of Environment Controlled Agricultural Engineering in Huang-Huai-Hai Region, Ministry of Agriculture, Beijing, China; ^4^College of Horticulture, Henan Agricultural University, Zhengzhou, China

**Keywords:** abscisic acid, evaporative demand, mesophyll conductance, plant water status, stomatal conductance

## Abstract

The atmospheric vapour pressure deficit (VPD) has been demonstrated to be a significant environmental factor inducing plant water stress and affecting plant photosynthetic productivity. Despite this, the rate-limiting step for photosynthesis under varying VPD is still unclear. In the present study, tomato plants were cultivated under two contrasting VPD levels: high VPD (3–5 kPa) and low VPD (0.5–1.5 kPa). The effect of long-term acclimation on the short-term rapid VPD response was examined across VPD ranging from 0.5 to 4.5 kPa. Quantitative photosynthetic limitation analysis across the VPD range was performed by combining gas exchange and chlorophyll fluorescence. The potential role of abscisic acid (ABA) in mediating photosynthetic carbon dioxide (CO_2_) uptake across a series of VPD was evaluated by physiological and transcriptomic analyses. The rate-limiting step for photosynthetic CO_2_ utilisation varied with VPD elevation in tomato plants. Under low VPD conditions, stomatal and mesophyll conductance was sufficiently high for CO_2_ transport. With VPD elevation, plant water stress was gradually pronounced and triggered rapid ABA biosynthesis. The contribution of stomatal and mesophyll limitation to photosynthesis gradually increased with an increase in the VPD. Consequently, the low CO_2_ availability inside chloroplasts substantially constrained photosynthesis under high VPD conditions. The foliar ABA content was negatively correlated with stomatal and mesophyll conductance for CO_2_ diffusion. Transcriptomic and physiological analyses revealed that ABA was potentially involved in mediating water transport and photosynthetic CO_2_ uptake in response to VPD variation. The present study provided new insights into the underlying mechanism of photosynthetic depression under high VPD stress.

## Introduction

Carbon dioxide (CO_2_) is significant for plant photosynthesis, growth, and yield production. Although CO_2_ fertilisation and globally elevated trends are expected to improve crop photosynthesis and yield, large evidence has shown that the magnitude of such enhancement is constrained by other climate change-derived phenomena, such as more extreme and frequent environmental stress (Norby, 2002). The bottlenecks constraining the CO_2_ utilisation efficiency are limited CO_2_ acquisition and assimilation. It has been recognised that CO_2_ movement and carbon fixation are regulated by environmental factors. There is increasing evidence from physiology and crop production that high vapour pressure deficit (VPD) induces plant water stress and inhibits photosynthetic productivity (Lu et al., 2015; Zhang et al., 2015). Few previous studies have quantitatively addressed the components of photosynthetic limitation across a series of VPD. The rate-limiting step for photosynthetic CO_2_ transport and utilisation under different VPD conditions was highly uncertain. A quantitative limitation analysis consisting of stomatal, mesophyll, and biochemical limitations is essential to reveal the underlying mechanism by which the VPD affects the photosynthetic process.

Photosynthetic CO_2_ uptake and transport are constrained by a series of resistances, which have been simplified into stomatal and mesophyll resistance (Tholen and Zhu, 2011). Guard cells of stomata are the first barrier for gas exchange and modulate photosynthetic CO_2_ uptake and transpiration (Lawson and Blatt, 2014). Large evidence has shown that CO_2_ movement from the substomatal cavity to the carbon fixation site is constrained by great mesophyll resistance (Niinemets et al., 2009; von Caemmerer and Evans, 2010; Flexas et al., 2012; Kaldenhoff, 2012; Sharkey, 2012; Li et al., 2019b). In addition to stomatal resistance, mesophyll resistance also substantially constrains the photosynthetic rate, especially for C_3_ plants. Environmental fluctuations are thought to profoundly affect CO_2_ uptake and transport. Leaf anatomical properties determine the maximum potential conductance for gas or liquid phase diffusion. Some studies attributed photosynthetic limitations to anatomical adaptations under high VPD stress, such as reduced stomatal size, stomatal density, vein density, and mesophyll surface area (Fanourakis et al., 2016, 2020; Du et al., 2019). CO_2_ uptake and water loss share some common pathways, such as stomatal and intercellular spaces. Anatomical adaptations prevent excessive water loss, which simultaneously increases diffusion resistance for CO_2_ uptake. In addition to the anatomical determination over long-term adaptations, much evidence has shown that stomatal and mesophyll conductance respond rapidly and sensitively to external environmental variation (Xiong et al., 2015; Li et al., 2019a,b). The field and greenhouse VPD fluctuate dramatically over the diurnal course, which significantly affects the photosynthetic process. However, less attention has been given to reveal the mechanism of the rapid response of CO_2_ diffusion conductance across a series of VPD.

It has been widely reported that the plant hormone abscisic acid (ABA) is involved in various abiotic stresses and acts as a signalling molecule in response to drought, salinization, heat, and so on (Fang et al., 2019). Cellular ABA accumulation is an important dehydration-sensing and water balance-maintaining mechanism, which has special implications in stomatal closure and the decline of hydraulic conductance (Sack et al., 2018). ABA prevents excessive water loss and enhances crop drought tolerance by signalling pathways. As a C_3_ plant species, the photosynthetic and yield potential of tomato plants is greatly limited by the low CO_2_ availability inside chloroplasts. The excessive evaporative demand under high VPD exceeds root water uptake capacity and triggers plant water deficit in tomato plants, which contributes to photosynthetic depression and yield loss (Zhang et al., 2017, 2018; Li et al., 2019b). We hypothesised that ABA plays a significant role in preventing transpiration under high VPD-induced plant water deficit, which simultaneously constrains photosynthetic CO_2_ uptake and acquisition. Identifying the rate-limiting step for photosynthetic CO_2_ acquisition under contrasting VPD and revealing the mechanism has significant implications for both basic plant sciences and crop production.

To investigate the effect of long-term acclimation on the short-term rapid VPD response, the implications of leaf anatomical properties and ABA in modulating CO_2_ transport across a series of VPD ranges were addressed by physiological and transcriptomic analyses. Three questions were addressed in the present study: (1) How did stomatal and mesophyll conductance tune with the VPD? (2) How did the contribution of stomatal and mesophyll limitation to photosynthesis vary with the VPD? (3) How did ABA tune with the VPD and correlate with stomatal and mesophyll conductance?

## Materials and Methods

### Plant Materials and Growth Conditions

The experiment was conducted in two environmentally controlled greenhouses with the same characteristics (15 m in length, 10 m in width, and 3.5 m in height, north-south oriented) under spring-summer climatic conditions from May to August 2018. Two widely grown tomato cultivars (JinPeng NO.1, CV1, JinPeng & Co., Ltd., China; FenGuan, CV2, ZhongYa & Co., Ltd., China) with relatively distinct VPD responses were examined (Du et al., 2020). Seeds were sown in plugs for germination and transplanted at the four-leaf stage to 4.5 L plastic pots containing the same amount of organic substrate and perlite mixture in a 3:1 proportion (v/v). Soil moisture was maintained at ~90% container capacity according to a previous method (Zhang et al., 2017). Plants were periodically trimmed to maintain rapid vegetative growth throughout experiments. Plants were grown in two environmentally controlled greenhouses and maintained under the same growth conditions but contrasting VPD. A high VPD was achieved in a natural greenhouse environment, with a VPD of ~3–5 kPa around midday, while low VPD was maintained in the range of.5–1.5 kPa by humidification. A high-pressure micro-fog system was activated when the VPD exceeded the target values, and the characteristics of the system were described in detail in a previous study by Zhang et al. (2018). The average daily meteorological data inside the greenhouse during the growth period were ~maintained at a temperature of 20–32°C, relative humidity of 50–75%, and photosynthetically active radiation of 45–65 Wm^−2^.

The effects of VPD perturbations on leaf photosynthetic performance and plant water status were investigated ~50 days after treatments. Afterward, 15 uniform plants from each treatment were selected as samples and transferred to growth cabinets in the evening prior to photosynthetic measurements. The light and temperature of the growth cabinets were controlled steadily at normal levels throughout the experiment.

### Leaf Gas Exchange and Chlorophyll Fluorescence

Leaf gas exchange and chlorophyll fluorescence were measured simultaneously on healthy and expanded leaflets at the same nodes by portable gas exchange systems equipped with a leaf chamber fluorometer (LI-6400, Li-Cor, Inc., Lincoln, NE, USA). All portable gas exchange systems were enclosed in growth cabinets. The VPD inside cabinets and the leaf chamber was simultaneously controlled across a series gradient of 0.5, 1.5, 2.5, 3.5, and 4.5 kPa. The temperature, light, and CO_2_ concentrations were controlled at the following constant and steady conditions throughout the experiment: temperature of 28 ± 1°C; saturating photosynthetic photon flux density (PPFD) of 1,100 μmol m^−2^ s^−1^; CO_2_ concentration of 400 μmol mol^−1^. The VPD was increased stepwise across the gradients for at least 60 min until photosynthesis and the plant water status achieved a new steady state.

The curve of the photosynthetic rate (P_n_) vs. intercellular CO_2_ concentration (C_i_) was determined using a previous procedure (Li et al., 2019b), across the VPD range of 0.5–4.5 kPa. Briefly, a P_n_-C_i_ curve was generated by controlling the ambient CO_2_ concentration (C_a_) from 400 to 300, 200, 150, 100, and 50 μmol mol^−1^ and then increased to 400 μmol mol^−1^. After re-achieving a steady-state at 400 μmol mol^−1^, C_a_ was increased gradually from 400 μmol mol^−1^ to 1,200 μmol mol^−1^. The carboxylation efficiency (CE) was estimated according to linear regression of the P_n_-C_i_ curve in the range of C_a_ ≤ 200 μmol mol^−1^ (Sun et al., 2016). The maximum rate of Rubisco carboxylation capacity (V_cmax_) and maximal rate of electron transport (J_max_) were determined according to the FvCB model (Farquhar et al., 1980).

### Estimation of Photosynthetic CO_2_ Diffusion Conductance

Carbon dioxide diffuses *via* stomatal and mesophyll barriers in a series circuit, which was driven by the CO_2_ partial pressure gradient (Li et al., 2019b). Stomatal conductance for CO_2_ diffusion (g_sc_) was determined according to the water diffusion conductance (g_sw_) and the ratio between molecular diffusivities of water and CO_2_ in gas (Giuliani et al., 2013). The mesophyll conductance (g_m_) was estimated by the variable J method (Harley et al., 1992):


(1) $$\begin{array}{r} {\text{g}}_{m}=\frac{P_{n}}{C_{i}-\frac{\Gamma^{*}\left(J+8\left(P_{\text{n}}+R_{\text{d}}\right)\right)}{J-4\left(P_{\text{n}}+R_{\text{d}}\right)}} \end{array}$$

where P_n_ is the net photosynthetic rate and C_i_ is the intercellular CO_2_ concentration. P_n_ and C_i_ were measured by steady-state gas exchange. R_d_ is the mitochondrial respiration rate in the light, and Γ^*^ is the CO_2_ compensation point inside the chloroplast. R_d_ and Γ^*^ were calculated according to a previous study by Laisk and Oja (1998). Briefly, P_n_-C_i_ curves were measured at two light intensities (75 and 500 μmol m^−2^ s^−1^) at CO_2_ concentrations of 30–120 μmol mol^−1^. Γ^*^ (x-axis) and R_d_ (y-axis) were derived according to the intersection point of the P_n_-C_i_ curves. J is the electron transport rate, which was calculated as described by a previous study (Tomas et al., 2013).

According to the series circuit, the total CO_2_ diffusion resistance (1/g_tot_) can be determined as 1/g_tot_ = 1/g_s_ + 1/g_m_ (Niinemets et al., 2009). Therefore, g_tot_ can be determined as:


(2) $$\begin{array}{r} g_{tot}=\frac{\text{1}}{\text{1/}g_{s}+\text{1/}g_{m}} \end{array}$$

### Partitioning of the Photosynthetic Limitation

The photosynthetic limitation was divided into the components of stomatal limitation (L_s_), mesophyll limitation (L_m_), and biochemical limitation (L_m_). The proportions of individual components imposed on photosynthesis were determined as follows (Muir et al., 2014; Li et al., 2019b):


(3) $$\begin{array}{r} L_{s}=\frac{g_{tot}/g_{s}\times \partial A/\partial C_{c}}{g_{tot}+\partial A/\partial C_{c}}\\ L_{m}=\frac{g_{tot}/g_{m}\times \partial A/\partial C_{c}}{g_{tot}+\partial A/\partial C_{c}}\\ L_{b}=\frac{g_{tot}}{g_{tot}+\partial A/\partial C_{c}} \end{array}$$

By definition, L_s_ + L_m_ + L_b_ =1; ∂*A*/∂*C*_c_ was determined as the slope of the P_n_-C_c_ curves at CO_2_ concentrations of 40–110 μmol mol^−1^.

### Determination of the Plant Water Status

Once photosynthetic measurements were completed, the adjacent leaflets were harvested for the determination of the water status of the plant. The leaf water potential (Ψ_leaf_) was measured by a pressure chamber (PMS-1000, PMS Instruments Inc., Corvallis, OR, USA). An extra test was performed where Ψ_leaf_ of adjacent leaflets was compared, and no differences in Ψ_leaf_ were detected between two adjacent leaflets. Some plants were kept in dark conditions for ~8–10 h for the determination of the soil water potential (Ψ_soil_) (Tsuda and Tyree, 2000). Since water movement was ~zero under dark conditions, Ψ_soil_ remained relatively constant and can be assumed to equal the xylem pressure potential of leaves under dark conditions.

### Leaf Morphology

After determination of the plant water status, the leaflet area was measured by a leaf area metre. The leaflet samples were dried at 80°C in an oven to a constant dry mass and weighed. The leaf mass area (LMA) was determined as the ratio of leaf dry mass to leaf area.

### Leaf ABA Concentration

After reaching the steady-state of photosynthesis, leaflets were harvested for ABA detection and transcriptome sequencing. Phytohormone contents were determined by a liquid chromatography electrospray ionisation tandem mass spectrometry (LC-ESI-MS/MS) system (HPLC, Shim-pack UFLC SHIMADZU CBM30A system, www.shimadzu.com.cn/; MS, Applied Biosystems 6500 Triple Quadrupole, www.appliedbiosystems.com.cn/). Briefly, the leaflets for photosynthetic measurements were harvested and frozen in liquid nitrogen. The samples were extracted with methanol/water/formic acid and filtered before LC-MS/MS analysis. The detailed protocol was described on MetWare (http://www.metware.cn/) based on the AB Sciex QTRAP 6500 LC-MS/MS platform. Samples were detected with three biological replicates.

### RNA Extraction and Transcriptome Sequencing

Total RNA was extracted from leaflet samples for transcriptome sequencing, according to a previous study (Zhang et al., 2019b). Sequencing libraries were constructed using the Ultra^TM^ RNA Library Prep Kit for Illumina (NEB, USA) according to the instructions of the manufacturer. The detailed protocol was described in a previous study, which was briefly described in a simplified diagram.

### Sequencing Data Analysis

The clean data were obtained by processing the raw data through in-house Perl scripts. The low-quality data and sequencing adapters were trimmed. The fragments per kilobase of transcript per million fragments mapped (FPKM) was calculated based on gene length and read counts. Differentially expressed genes (DEGs) were assigned according to the adjusted *P* < 0.05. Gene ontology (GO) enrichment was determined by submitting DEGs to the GO database to classify the genes. Kyoto Encyclopaedia of Genes and Genomes (KEGG; https://www.genome.jp/kegg) was used to perform pathway enrichment analysis. Terms with corrected *P* < 0.05 were identified as significantly enriched by DEGs. To confirm the reliability of transcriptome sequencing, 10 candidates expressed genes in RNA-seq were simultaneously evaluated by qRT–PC analysis. The qRT–PCR values were linearly correlated with the RNA-seq FPKM values (*P* < 0.001).

### Statistical Analyses

All statistical analyses were performed using SPSS 19. One-way ANOVA was used to determine the significant difference of average values according to Tukey's test (*P* < 0.05). Regression analysis was performed by Microsoft Excel.

## Results

### Effect of VPD on Water Transport Forces Along the Soil-Plant-Atmospheric Continuum

**Figure 1:**
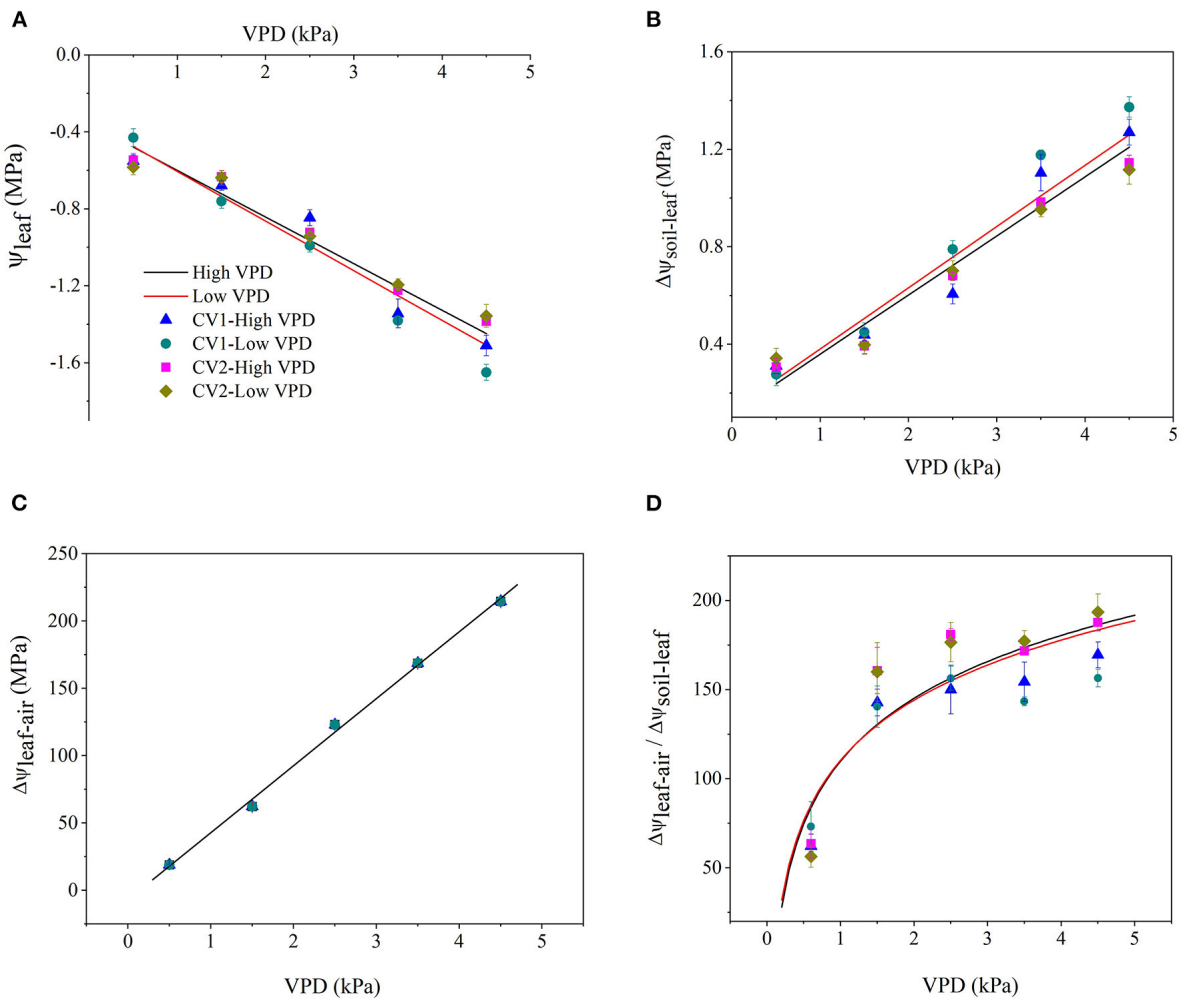
Effect of the vapour pressure deficit (VPD) on the spatial distribution of the water potential and driving force (ΔΨ) between two spatial positions. Values are the mean ± SE (*n* = 4~6 replicates). The regression lines shown are: **(A)** HVPD, Ψ_leaf_ = −0.242 VPD −0.358, R^2^ = 0.92; LVPD, Ψ_leaf_ = −0.258 VPD −0.347, R^2^ = 0.93. **(B)** HVPD, ΔΨ_soil−leaf_ = 0.242 VPD + 0.118, R^2^ = 0.94; LVPD, ΔΨ_soil−leaf_ = 0.251 VPD + 0.13, R^2^ = 0.93. **(C)** HVPD, ΔΨ_leaf−air_ = 49.8 VPD −6.86, R^2^ = 0.98; LVPD, ΔΨ_leaf−air_ = 49.8 VPD−6.86, R^2^ = 0.98. **(D)** HVPD, ΔΨ_leaf−air_/ΔΨ_soil−leaf_ = 50.82 ln (VPD) + 109.95, R^2^ = 0.85; LVPD, ΔΨ_leaf−air_/ΔΨ_soil−leaf_ = 48.63 ln (VPD) + 110.41, R^2^ = 0.8.

Vapour pressure deficit significantly affected the distribution of water potential along the soil-plant-atmospheric pathway (Figure 1). Atmospheric evaporative demand increased with VPD elevation, which triggered plant water stress and a linear decline in the leaf water potential (Figure 1A). With VPD elevation, the drawdown of Ψ_leaf_ in high-VPD-grown plants was less than that in low-VPD-treated plants according to the slope of linear regression (Figure 1A). The driving force for passive water flow between the soil and leaf (ΔΨ _soil−leaf_) increased with the VPD, and the magnitude of the increase was greater in low-VPD-grown plants than high-VPD-grown plants (Figure 1B). Since Ψ_leaf_ was negligible compared with the large negative air potential, the water driving force at the leaf-air boundary (ΔΨ_leaf−air_) increased dramatically with an increase in the VPD (Figure 1C). The magnitude of the increase in ΔΨ_leaf−air_ was considerably greater than that of ΔΨ_soil−leaf_, and the difference between ΔΨ_leaf−air_ and ΔΨ _soil−leaf_ was enlarged with the VPD: the ratio of ΔΨ_leaf−air_ to ΔΨ_soil−leaf_ increased logarithmically from ~50 at 0.5 kPa to 150 at 1.5 kPa and then maintained at a steady level (Figure 1D). The statistical analyses of the plant water status are shown in Supplementary Table 1.

### Effect of VPD on the Photosynthetic Parameters of Tomato Plants

The photosynthetic rate responded to CO_2_ elevation in similar patterns regardless of cultivar and VPD growth conditions: the photosynthetic rate rose rapidly across low CO_2_ concentrations and then reached a steady state (Supplementary Figure 1). The maximum steady-state photosynthetic rate declined as the VPD increased from 0.5 to 4.5 kPa (Supplementary Figure 1). The maximum carboxylation rate (V_cmax_), maximum electron transport rate (J_max_), and CE declined linearly with VPD elevation (Supplementary Figure 2). The drawdown of V_cmax_, J_max_, and CE with VPD elevation was moderated in high-VDP-grown plants compared with low-VPD-grown plants according to the slope of linear regression (Supplementary Figure 2). The statistical analyses of photosynthetic parameters across VPD ranges are shown in Supplementary Table 2.

### Effect of VPD on the Photosynthetic CO_2_ Uptake and Transport

**Figure 2:**
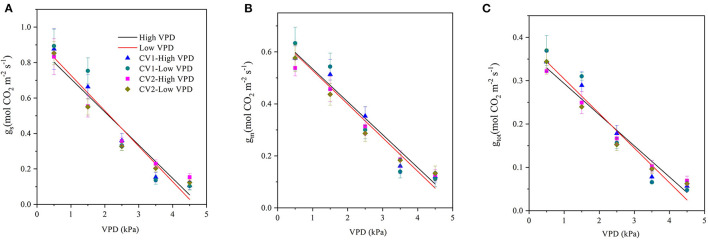
Effect of the VPD on the stomatal conductance (g_s_), mesophyll conductance (g_m_), and total conductance (g_tot_) for photosynthetic carbon dioxide (CO_2_) diffusion. Values are the mean ± SE (*n* = 4 replicates). The regression lines shown are: **(A)** HVPD, g_s_ = −0.187 VPD + 0.895, R^2^ = 0.94; LVPD, g_s_ = −0.200 VPD + 0.928, R^2^ = 0.92. **(B)** HVPD, g_m_ = −0.126 VPD + 0.661, R^2^ = 0.95; LVPD, g_m_ = −0.129 VPD + v0.658, R^2^ = 0.94. **(C)** HVPD, g_tot_ = −0.0719 VPD +0.365, R^2^ = 0.95; LVPD, g_tot_ = −0.0798 VPD + 0.384, R^2^ = 0.94.

The stomatal, mesophyll, and total conductance for CO_2_ diffusion decreased linearly with VPD elevation, regardless of the cultivar and VPD growth conditions (Figure 2). The magnitudes of drawdown in the stomatal, mesophyll, and total conductance were lower in high-VPD-grown plants than in low-VPD-grown plants for two cultivars (Figure 2).

**Figure 3:**
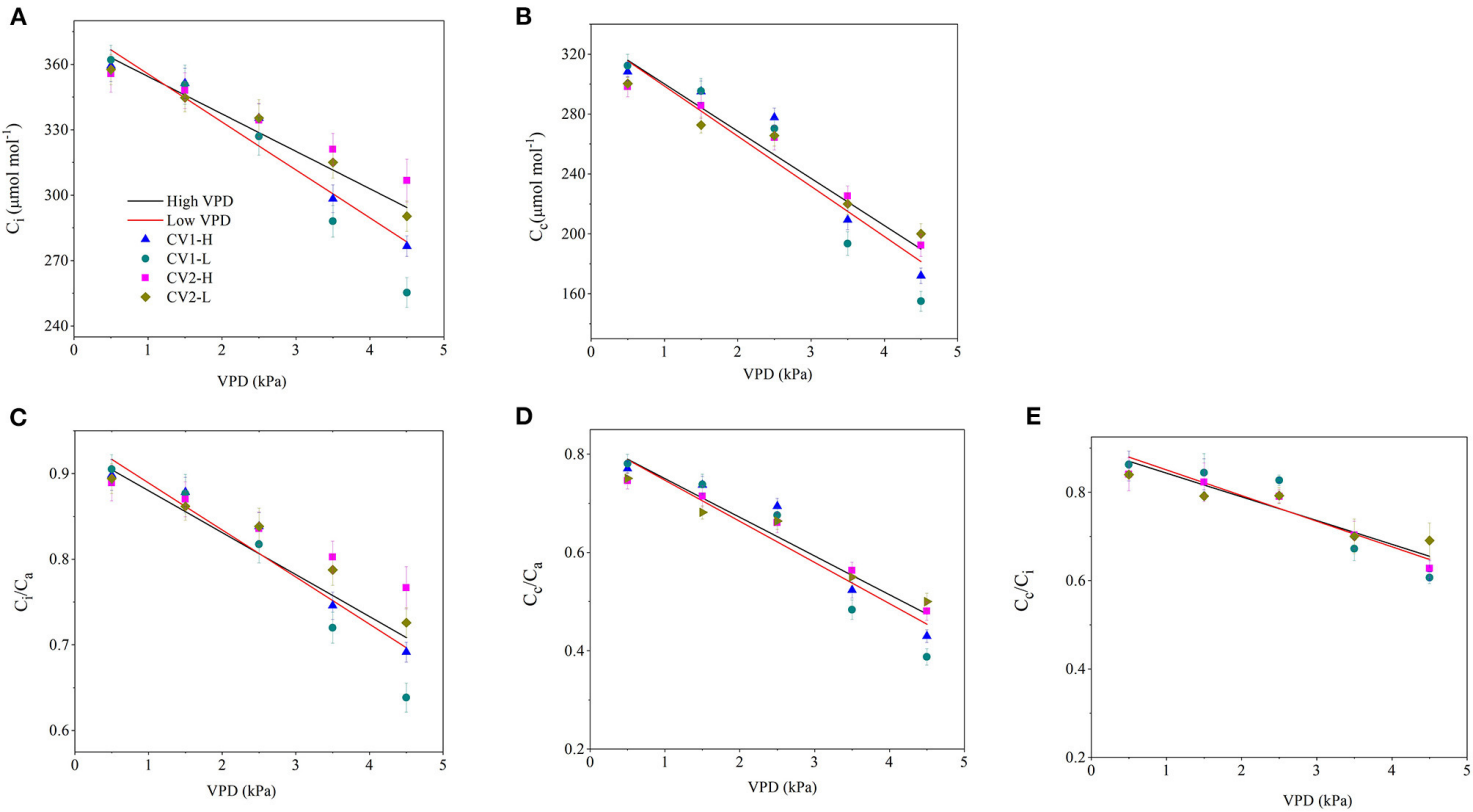
Effect of the VPD on the intracellular CO_2_ concentration [**(A)**; C_i_], carboxylation sites inside chloroplasts CO_2_ concentration [**(B)**; C_C_], the ratio of the intercellular to ambient CO_2_ concentration [**(C)**; C_i_/C_a_], the ratio of the chloroplast to ambient CO_2_ concentration [**(D)**; C_c_/C_a_] and the ratio of the chloroplast to intercellular CO_2_ concentration [**(E)**; C_c_/C_i_]. The regression lines shown are: **(A)** HVPD, C_i_ = −17.2 VPD + 371.6, R^2^ = 0.86; LVPD, C_i_ = −22 VPD + 377.6, R^2^ = 0.87. (B) HVPD, C_C_ = −31.5 VPD + 331.6, R^2^ = 0.91; LVPD, C_C_ = −33.5 VPD + 332.2, R^2^ = 0.88. **(C)** HVPD, C_i_/C_a_ = −0.0429 VPD + 0.93, R^2^ =0.86; LVPD, C_i_/C_a_= −0.055 VPD + 0.94, R^2^ = 0.87. **(D)** HVPD, C_c_/C_a_ = −0.0788VPD + 0.83, R^2^ = 0.92; LVPD, C_c_/C_a_ = −0.0837VPD + 0.83, R^2^ = 0.87. **(E)** HVPD, C_c_/C_i_ = −0.0537 VPD + 0.89, R^2^ = 0.82; LVPD, C_c_/C_i_ = −0.058VPD + 0.91, R^2^ = 0.89.

The CO_2_ concentration along the “source-path-sink” was reduced to different extents with VPD elevation (Figures 3A,B). The drawdowns of C_i_ and C_c_ caused by VPD elevation were relatively lower in high-VPD-grown plants than in low-VPD-grown plants (Figures 3A,B). Consequently, the CO_2_ transport efficiency of C_i_/C_a_, C_c_/C_a_ and C_c_/C_i_ decreased linearly with VPD elevation. The declining slopes of C_i_/C_a_, C_c_/C_a_, and C_c_/C_i_ vs. VPD were lower in high-VPD-grown plants than in low-VPD-grown plants (Figures 3C–E). The statistical analyses of CO_2_ concentrations along the “source-path-sink” across VPD ranges are shown in Supplementary Table 3.

### Partial Photosynthetic Limitation

**Figure 4:**
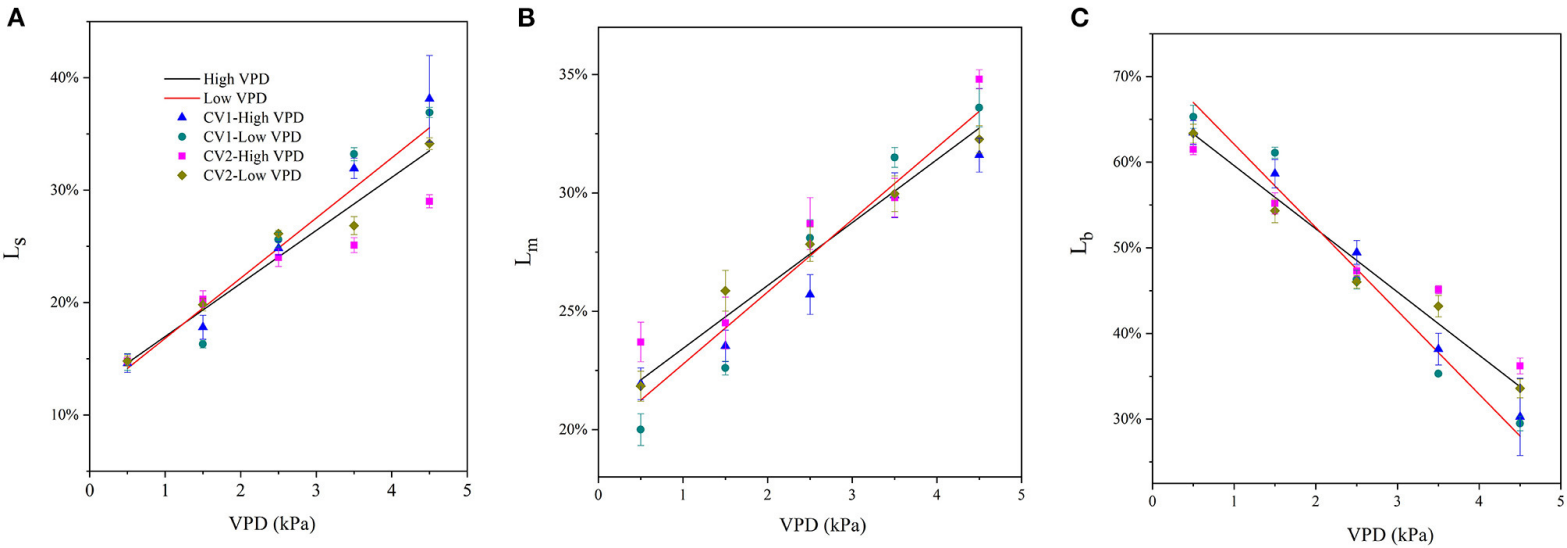
Quantitative limitation analysis comparing stomatal [**(A)**; L_s_], mesophyll [**(B)**; L_m_], and biochemical [**(C)**; L_b_] limitations imposed on the photosynthetic rate under varying VPD. The regression lines shown are: **(A)** HVPD, Ls = 0.0472 VPD + 0.122, R^2^ = 0.86; LVPD, Ls = 0.0535 VPD + 0.115, R^2^ = 0.89. **(B)** HVPD, L_m_ = 0.0266 VPD + 0.208, R^2^ = 0.88; LVPD, L_m_ = 0.0305 VPD + 0.197, R^2^ = 0.91.

The fractions of stomatal, mesophyll, and biochemical limitations imposed on photosynthesis varied with VPD elevation (Figure 4). Under low VPD conditions, the stomatal and mesophyll conductance for CO_2_ diffusion were high and imposed relatively minor limitations on photosynthesis. The stomatal and mesophyll conductance accounted for a low proportion of photosynthetic limitation, while biochemical carboxylation for carbon fixation was the most significant limitation for photosynthetic processes under low VPD conditions (Figure 4). The fraction of stomatal limitation increased linearly with the VPD, from ~15% at 0.5 kPa to 35% at 4.5 kPa (Figure 4A). A similar pattern was observed in the mesophyll limitation: the fraction of mesophyll limitation also increased linearly with VPD elevation, from ~23% at 0.5 kPa to 33% at 4.5 kPa (Figure 4B). The increments in the fractions of stomatal and mesophyll limitations tended to be less marked in high-VPD-grown plants. In contrast, the fraction of total limitations attributed to the biochemical limitation of carbon fixation gradually decreased linearly with VPD elevation, from ~65% at.5 kPa to 35% at 4.5 kPa (Figure 4C). The statistical analyses of stomatal, mesophyll, and biochemical limitation fractions across VPD ranges are shown in Supplementary Table 4.

**Figure 5:**
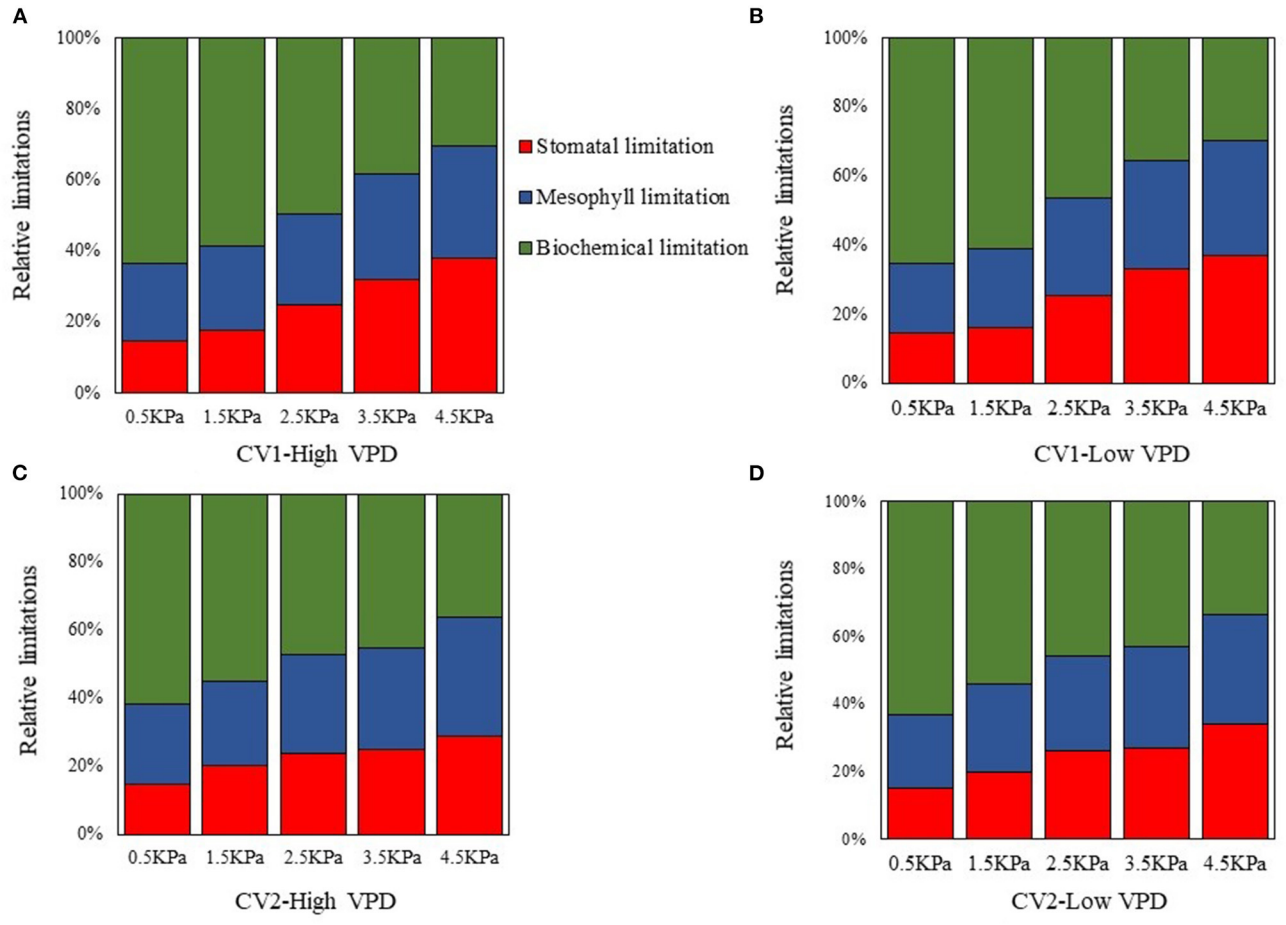
The dynamic changes in the relative proportions of individual components of photosynthetic limitations across VPD ranges: **(A)** CV1 grown under high VPD condition; **(B)** CV1 grown under low VPD condition; **(C)** CV2 grown under high VPD condition; and **(D)** CV2 grown under low VPD condition.

Biochemical limitation accounted for the greatest limitation on photosynthesis under low VPD conditions, regardless of the cultivar and growth conditions (Figure 5). The limitations that stomatal and mesophyll conductance imposed on photosynthesis gradually increased and predominated under high VPD stress (Figure 5). Diffusion limitations, i.e., the sum of the stomatal and mesophyll resistance, were the rate-limiting step for the photosynthetic process under high VPD conditions, which imposed the greatest limitation on photosynthesis in tomato plants (Figure 5).

### Correlations Among g_m_, g_s_, Leaf Water Status, and LMA

The mesophyll conductance was significantly and positively correlated with the stomatal conductance (Supplementary Figure 3A). Meanwhile, the stomatal and mesophyll conductance for CO_2_ diffusion were closely linked to the leaf water status, wherein significant and positive correlations were found in the leaf water potential vs. the stomatal and mesophyll conductance (Supplementary Figures 3B,C). Acclimation to VPD modified leaf structural traits, wherein LMA tended to be slightly greater in high-VPD-grown plants than in low-VPD-grown plants (Supplementary Figure 4A). A significant and negative correlation between g_m_ and LMA was observed (Supplementary Figure 4B).

### Leaf ABA Concentration and Correlation With CO_2_ Diffusion Conductance

**Figure 6:**
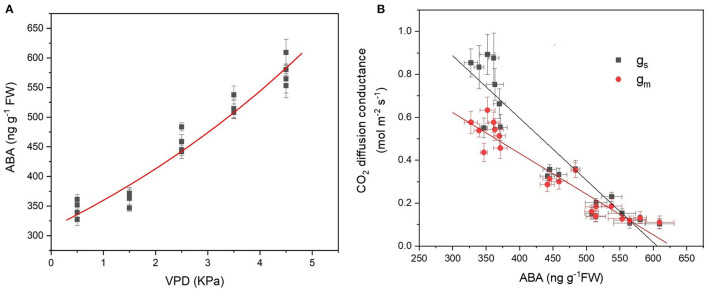
Leaf ABA content in response to the VPD **(A)** and its correlation with the CO_2_ diffusion conductance of g_s_ and g_m_ **(B)**. The regression lines shown are ABA = 301.3e^0.15VPD^, R^2^ = 0.96, *P* < 0.01; g_s_ = −0.0029 ABA + 1.76, R^2^ = 0.85, *P* < 0.01; g_m_ = −0.0019 ABA + 1.19, R^2^ = 0.88, *P* < 0.01.

With VPD elevation, the foliar ABA content increased exponentially (Figure 6A). The leaf ABA content was linearly and negatively correlated with the CO_2_ diffusion conductance of g_s_ and g_m_ (Figure 6B). The slope of linear regression in g_s_ was more negative than that in g_m_, indicating that g_s_ was more sensitive to ABA in response to VPD stress (Figure 6B).

### Transcriptomic Analysis of Plant Response Across Series of VPD Ranges

**Figure 7:**
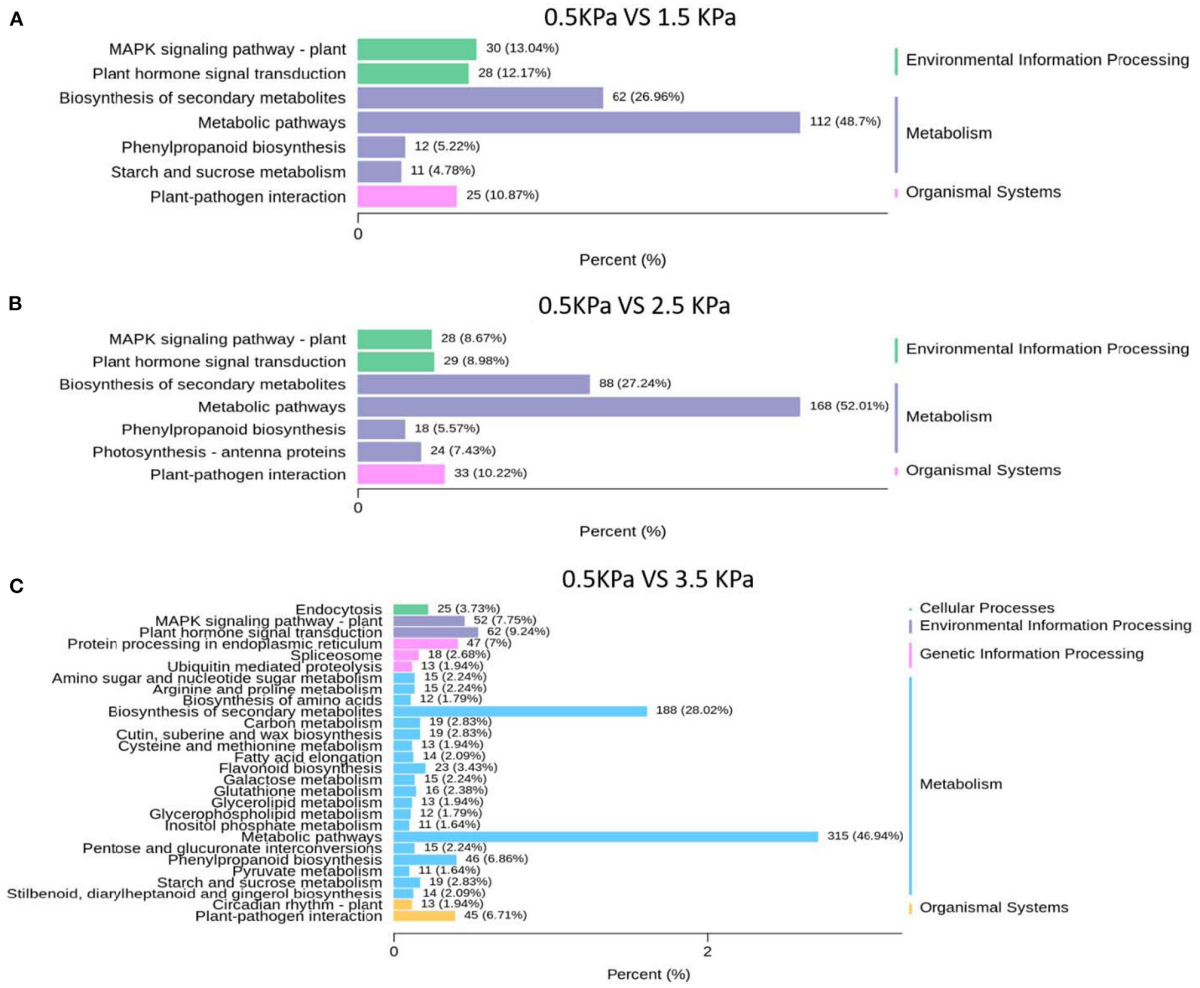
KEGG classification analysis of differentially expressed genes under different VPD conditions: **(A)** 0.5 kPa versus 1.5 kPa; **(B)** 0.5 kPa versus 2.5 kPa; and **(C)** 0.5 kPa versus 3.5 kPa.

Kyoto Encyclopaedia of Genes and Genomes analysis showed that physiological processes of “metabolic pathway” and “plant hormone signal transduction” were involved in the response to VPD and were potentially associated with ABA biosynthesis and signal transduction (Figure 7). “Metabolic pathway” was the dominant pathway in response to VPD elevation. “Plant hormone signal transduction” also exhibited a significant pathway in response to VPD stress in the range from 1.5 to 3.5 kPa (Figure 7).

**Figure 8:**
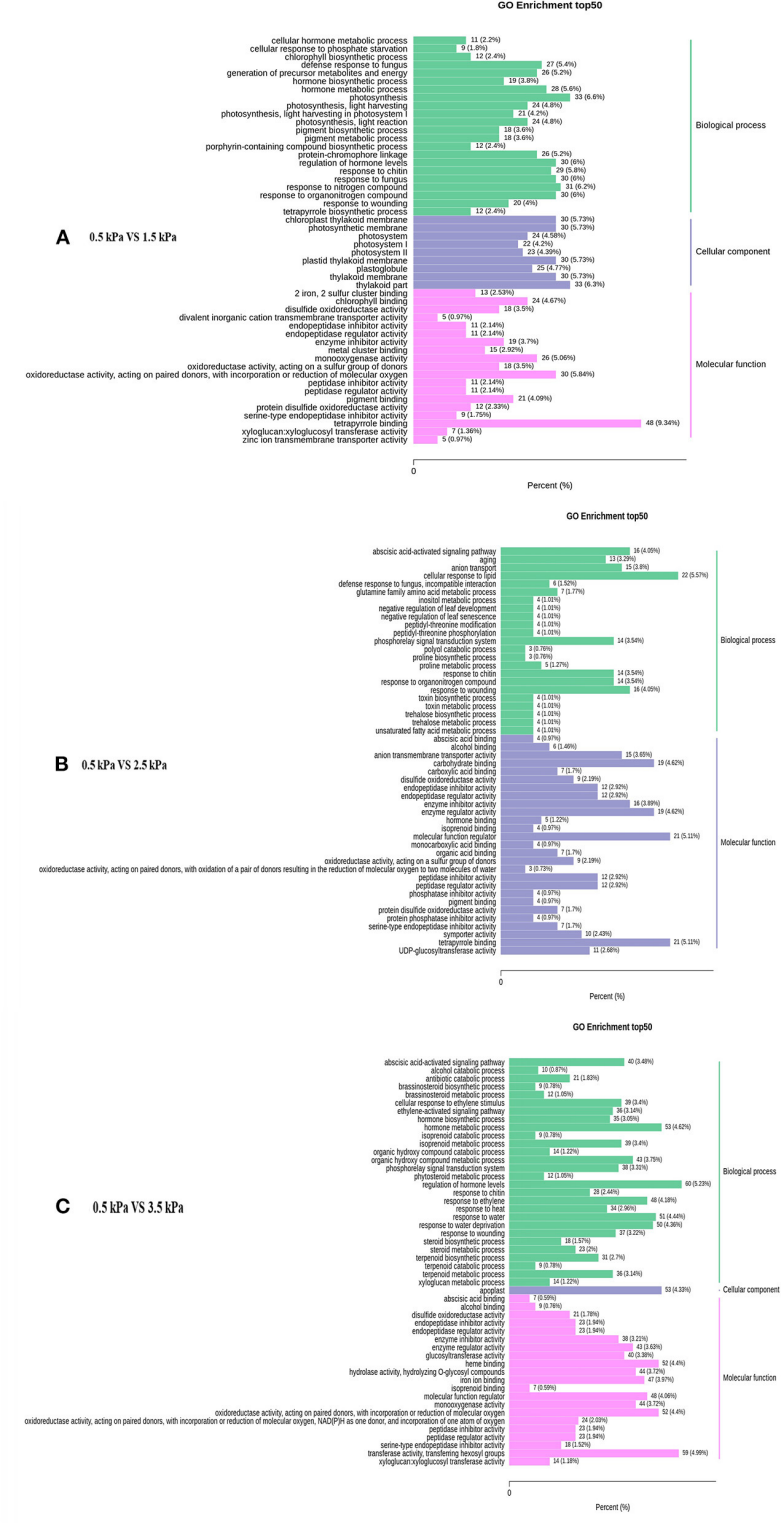
Top 50 enriched GO terms of the differentially expressed genes under different VPD conditions: **(A)** 0.5 kPa versus 1.5 kPa; **(B)** 0.5 kPa versus 2.5 kPa; and **(C)** 0.5 kPa versus 3.5 kPa.

**Figure 9:**
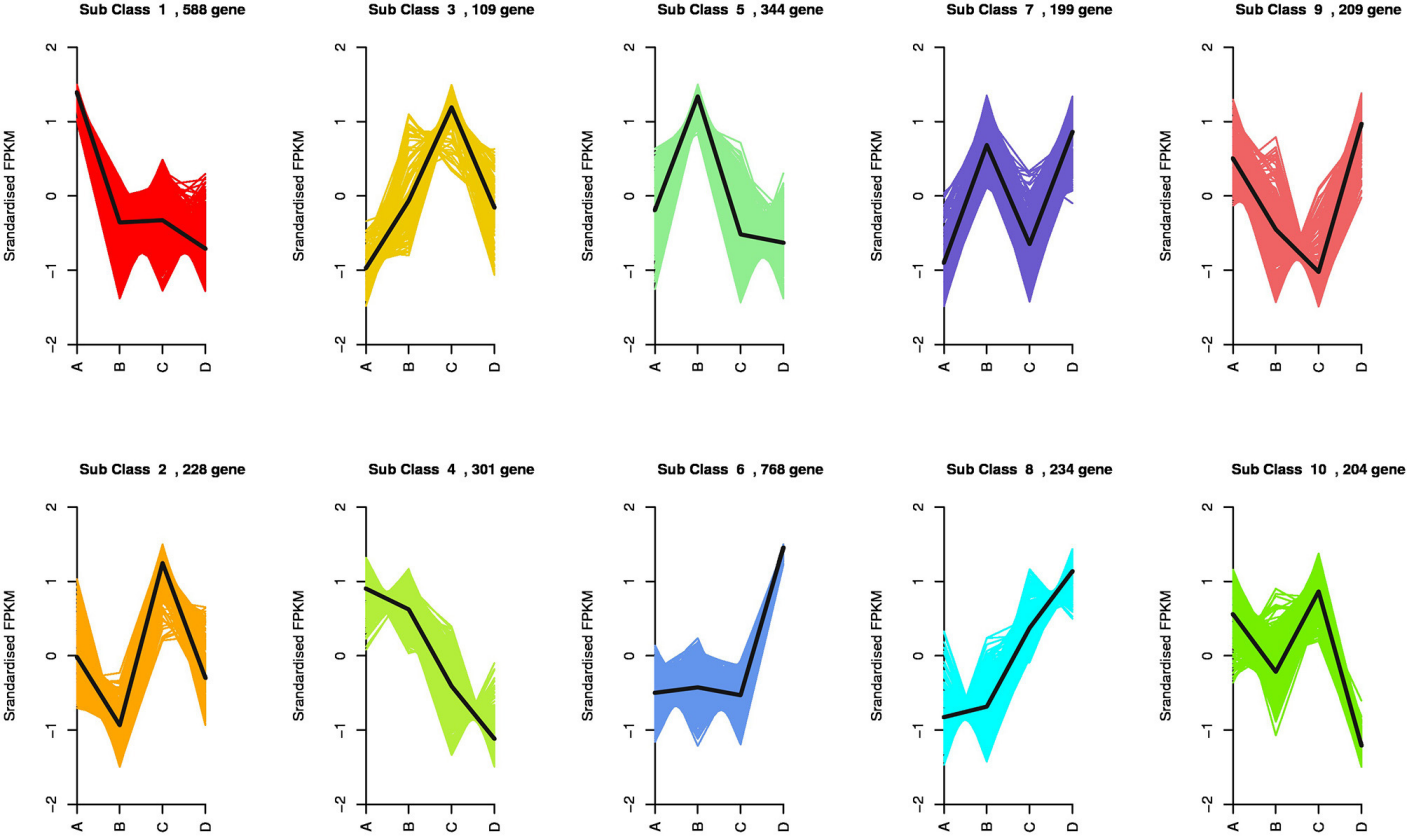
Expression model of DEGs across a series of VPD ranges. A, B, C, and D in the X-axis in the figures represent 0.5, 1.5, 2.5, and 3.5 kPa, respectively.

The enriched genes in the comparison between different VPD treatments were annotated in three main GO categories: biological process, cellular component, and molecular function. A great difference in the top 50 GO enrichment terms was observed between different VPD conditions (Figure 8). In the comparison of 0 with 1.5 kPa and 0.5 kPa with 3.5 kPa, the “ABA-activated signalling pathway” was significantly enriched and was involved in the VPD response (Figures 9A,C). In the comparison of 0.5 kPa with. 2.5 kPa, ABA was potentially involved according to the enriched terms of “cellular hormone metabolic process,” “hormone biosynthetic process,” and “hormone metabolic process”. Gene expression with rising VPD can be classified into 10 patterns according to K-means analysis (Figure 9). Gene expression patterns were mostly classified into the pattern of “Subclass 6” with 768 genes, wherein gene expression remained relatively stable under mild VPD stress and increased dramatically under high VPD water stress (Figure 9). The genes associated with ABA biosynthesis and signal transduction followed different patterns.

## Discussion

The present study assessed the rate-limiting step for tomato plant photosynthesis across a series of VPD ranges and evaluated ABA-mediated regulatory mechanisms according to physiological and transcriptomic analyses. The key rate-limiting step for photosynthetic performance varied with the VPD: under low VPD conditions, stomatal, and mesophyll conductance was high for efficient CO_2_ transport, which facilitated sufficiently high CO_2_ availability inside chloroplasts for carbon fixation (Figures 2, 3). With VPD elevation, the stomatal and mesophyll conductance for CO_2_ transport declined gradually. Consequently, photosynthesis was substantially constrained by the low chloroplast CO_2_ concentration under high VPD conditions (Figure 3). Therefore, the CO_2_ diffusion limitation in a series of stomatal and mesophyll resistances was the key rate-limiting step for photosynthesis under high VPD conditions (Figure 4). In addition to anatomical determination, ABA accumulation and signal transduction were involved in maintaining the water balance in response to VPD. ABA accumulation was negatively correlated with CO_2_ diffusion conductance (Figure 6). Three steps were involved in the potential mechanism accounting for the increased limitation of stomatal and mesophyll conductance imposed on tomato plant photosynthesis with VPD elevation (Supplementary Figure 5): (I) VPD elevation caused plant water stress by disrupting the mass balance between the soil water supply and atmospheric evaporative demand; (II) plants maintained the water balance by regulating ABA accumulation and signal transduction in response to high VPD stress; (III) ABA in combination with leaf anatomical adaptation modulated CO_2_ uptake and transport.

### VPD Elevation Triggers Plant Water Stress by Disrupting the Mass Balance Between Soil Water Supply and Atmospheric Evaporative Demand

Passive water movement was driven by the gradient of free energy along the soil-plant-atmospheric continuum, which could be quantified as the gradient in water potential in the liquid phase. Water movement at the leaf-air boundary in the gas phase was driven by the difference in the VPD. Based on physical principles, excessive air desiccation triggered a high VPD and great negative air-water potential. Δψ_leaf−air_ was substantially >Δψ_soil−leaf_, which drove transpiration. The substantial difference between Δψ_leaf−air_ and Δψ_soil−leaf_ was logarithmically enlarged with an increase in the VPD (Figure 1). Quantitatively, the atmospheric driving force at the leaf-air boundary could be >100-fold larger than the soil-leaf component under high VPD conditions (Figure 1). The great asymmetry between the atmospheric evaporative demand and soil water supply triggered disruption in the water balance despite plants being well irrigated. Root water uptake and supply were inadequate to keep pace with the great atmospheric driving force under high VPD conditions, which consequently triggered leaf dehydration and decline in water potential. Therefore, the VPD is a crucial external stimulus moving water through a soil-plant-environment continuum. VPD fluctuates dramatically over the diurnal course in crop production, especially for greenhouse cultivation. Soil moisture is relatively stable over the short term compared with the VPD (Caldeira et al., 2014). Plant-water relations are regulated to a greater extent by the VPD and to a lesser extent by soil moisture. Similar to soil drought, VPD-induced plant water stress is also an important factor triggering photosynthetic depression.

### Plants Maintain the Water Balance by Regulating ABA Accumulation and Signal Transduction in Response to High VPD Stress

The stoma is the “gatekeeper” for the exchange of water vapour and CO_2_. Guard cells surrounding the stomatal pore respond to perturbations of the soil-plant-atmospheric hydraulic continuum, which is putatively transduced into stomatal movements by feedback and feedforward mechanisms (Buckley, 2005, 2017, 2019). Stomatal control of transpired water loss is critical for sustaining physiological processes, such as leaf water status and photosynthetic CO_2_ uptake. It has been recognised that plants respond to drought by closing guard cells to prevent the development of water dehydration in plant tissues (Novick et al., 2016). In the present study, the atmospheric driving force was an order of magnitude greater than the water supply, which led to a great dissymmetry between the water supply and evaporative demand. The dissymmetry between the water supply and evaporative demand triggered declines in the leaf water potential and stomatal closure. However, the mechanism of VPD-triggered stomatal closure is still uncertain and is a “black box” (Buckley, 2016). Some hypotheses hold that stomatal closure in angiosperms under high VPD conditions is an active process that is regulated by hormonal and hydraulic signals (Merilo et al., 2018; Pantin and Blatt, 2018). The plant stress hormone ABA is continuously produced and delivered with a transpiration stream to guard cells (Qiu et al., 2017; Merilo et al., 2018). In the present study, leaf ABA rapidly accumulated with the rise in the VPD. Transcriptome analysis suggested that ABA biosynthesis and signal transduction were potentially involved in the response to the VPD. Based on the theory of ion channel-mediated guard cell signal transduction (Julian et al., 2001), hypothetical mechanisms of the ABA-mediated stomatal closure response to high VPD-induced water stress are proposed in Supplementary Figure 6. Ions and water flowed into guard cells under low VPD conditions and sustained turgor for stomatal openness. Under high VPD-induced water stress, ABA rapidly accumulated and promoted stomatal closure by altering ion channel activities.

Although stomatal closure prevented excess water loss to maintain physiological processes by passive or active mechanisms, closed “gatekeepers” simultaneously increased stomatal resistance for photosynthetic CO_2_ uptake from air to intercellular. The intercellular CO_2_ concentration was gradually reduced with VPD elevation (Figure 3). Consequently, the stomatal limitation imposed on photosynthesis gradually became pronounced with VPD elevation (Figure 4). The declines in the leaf water potential and stomatal conductance with VPD elevation were less marked in high-VPD-grown plants in the present research. The distinct response to the VPD is potentially modulated by physiological acclimation to growth conditions (Fanourakis et al., 2019). In contrary to the previous study, no significant differences were observed in the photosynthetic parameters across VPD ranges between the two examined cultivars. The distinct responses of two cultivars between the previous and present study were potentially caused by the examined VPD conditions. The dynamic VPD response of the present and previous studies were performed under cabinets and greenhouse conditions, respectively.

### Anatomical Properties and ABA Modulate Mesophyll Conductance Under Contrasting VPD Conditions

In addition to the first barrier of stomata, CO_2_ movement from intercellular to carboxylation sites is constrained by mesophyll resistance. The present study demonstrated that mesophyll resistance was a significant component of diffusion resistance from air to Rubisco in tomato plants. A strong positive correlation between the mesophyll and stomatal conductance was observed among treatments (Figure 6). Similar to the stomatal conductance, the mesophyll conductance of tomato plants was also linearly reduced with VPD elevation (Figure 2). Under low VPD conditions, stomatal conductance coupled with mesophyll conductance was high for efficient CO_2_ transport to carboxylation sites within chloroplasts. High diffusion conductance facilitated high chloroplast CO_2_ concentrations for carbon fixation (Figure 3). With VPD elevation, the CO_2_ concentration inside chloroplasts was substantially reduced under high VPD conditions. The limitation of mesophyll conductance imposed on photosynthesis gradually dominated with VPD elevation (Figure 5). Leaf anatomical traits from the substomatal cavity to the carbon fixation site determine the maximum potential of mesophyll conductance (Muir et al., 2014; Xiong et al., 2017; Earles et al., 2018; Han et al., 2018; Carriqui et al., 2019). The LMA is a composite of underlying traits such as the lamina thickness, mesophyll thickness, cell wall thickness, cell shape, and bulk leaf density, which anatomically regulate the mesophyll conductance (Muir et al., 2014). The LMA determines the upper limit on mesophyll conductance. Meanwhile, the LMA is closely linked to abiotic stress tolerance (Xiong and Flexas, 2018; Xiong et al., 2018). Generally, a higher LMA is a good indicator of greater stress tolerance. In the present study, the LMA of high-VPD-grown plants was lower but higher than that of low-VPD-grown plants (Supplementary Figure 4). Long-term acclimation to a high VPD facilitates enhanced drought tolerance to prevent dehydration by regulating the leaf thickness, cuticular permeability, stomatal morphology, and other anatomical features (Fanourakis et al., 2016, 2020). Long-term exposure to a VPD also affects stomatal sensitivity and morphological features such as the stomatal size, density, index, and spacing, which consequently modulate transpired water loss (Fanourakis et al., 2016, 2020). As mentioned above, root water uptake and supply are inadequate to keep pacing with the great atmospheric driving force under high VPD conditions. A higher LMA indicated dense structural traits, which buffered cellular transpired water loss and prevented leaf tissue dehydration under high VPD conditions. However, CO_2_ and water transport share pathways through the mesophyll cell walls and perhaps plasma membranes within leaves (Barbour, 2017; Groszmann et al., 2017; Zhao et al., 2017; Drake et al., 2019). Although dense structural traits improved drought tolerance, the resistance of CO_2_ diffusion was simultaneously increased. The LMA was negatively correlated with mesophyll conductance in the present study, which is consistent with previous studies (Hassiotou et al., 2009).

In addition to anatomical determinations, biochemical regulations such as ABA, carbonic anhydrase, and aquaporin facilitate rapid mesophyll conductance responses to short-term changing environmental factors (Momayyezi et al., 2020). The mesophyll conductance is negatively correlated with the leaf ABA content in tomato plants, which is in accordance with a previous study (Sorrentino et al., 2016; Qiu et al., 2017). The foliar ABA content rapidly increased upon long-term and short-term exposure to a high VPD, which is in accordance with a previous study (McAdam and Brodribb, 2016). However, the ABA-mediated regulatory mechanism has rarely been reported. CO_2_ entering from intercellular to carboxylation sites inside chloroplasts must pass through plasma membranes. The resistance of transport across the membrane accounts for a great proportion of the mesophyll resistance. It is now well established that aquaporins function as water pores for water transport across membranes and play significant roles in maintaining water homeostasis in response to drought and salinity (Qian et al., 2015; Zhang et al., 2019a). There is increasing evidence that some specific aquaporins (which localise to the plasma membrane and chloroplast inner envelope membrane) are permeable to CO_2_ and contribute to the mesophyll conductance (Uehlein et al., 2012; Groszmann et al., 2017; Zhao et al., 2017). Similar to guard cells, some *PIPs* pores mediate CO_2_ uptake and water transport across the plasma membrane (Zhang et al., 2021). Therefore, the specific *PIPs* potentially reconciled the trade-off between carbon gain and water loss in response to VPD-induced water stress. *PIPs* were sensitive to drought signals and responded rapidly to enclose gating and inhibit activity.

Gating is a general mechanism of membrane-mediated channels for controlling the permeability of water and CO_2_. Although the inhibition of *PIPs* channels prevents water loss under high VPD stress, CO_2_ uptake across the membrane is also restricted by the gating enclosure. ABA has been reported as a signal-inducing variation in the aquaporin content and activity (Fang et al., 2019). Therefore, VPD potentially modulated *PIPs* gating for CO_2_ and water permeability *via* ABA signalling, which contributed to mesophyll conductance and the photosynthetic rate.

## Conclusions

The present study revealed the rate-limiting step for photosynthetic CO_2_ utilisation under contrasting VPD conditions and proposed ABA-mediated regulatory mechanisms according to transcriptomic and physiological evidence. The photosynthetic performance of tomato plants was gradually constrained with VPD elevation. The key rate-limiting steps for photosynthetic performance varied with the rise in the VPD. With VPD elevation, plant water stress was gradually pronounced and triggered linear declines in the stomatal and mesophyll conductance. The contributions of stomatal and mesophyll limitations to photosynthesis increased gradually with VPD elevation. Consequently, the low CO_2_ availability inside chloroplasts substantially constrained photosynthesis under high VPD conditions. Leaf ABA accumulated rapidly with pronounced water stress under a high VPD and negatively correlated with the stomatal and mesophyll conductance for CO_2_ diffusion. Transcriptomic combined with physiological analyses revealed that ABA biosynthesis and signal transduction were potentially involved in mediating CO_2_ transport in response to the VPD.

## Data Availability Statement

The original contributions presented in the study are publicly available. This data can be found here: National Center for Biotechnology Information (NCBI) BioProject database under accession number PRJNA762604.

## Author Contributions

DZ, QL, and MW conceived and designed the experiments. PS, JL, and XL conducted the experiments. QD analysed the data and wrote the draft. All the authors reviewed and approved the manuscript.

## Funding

This project was supported by the National Natural Science Foundation of China (32102466), the Natural Science Foundation of Shandong Province (ZR2019BC035), and the Major Scientific Innovation Project of Shandong Province (2019JZZY010715).

## Conflict of Interest

The authors declare that the research was conducted in the absence of any commercial or financial relationships that could be construed as a potential conflict of interest.

## Publisher's Note

All claims expressed in this article are solely those of the authors and do not necessarily represent those of their affiliated organizations, or those of the publisher, the editors and the reviewers. Any product that may be evaluated in this article, or claim that may be made by its manufacturer, is not guaranteed or endorsed by the publisher.

## Abbreviations

VPD: vapour pressure deficit
Ψ_leaf_: leaf water potential
Ψ_soil_: soil water potential
Ψ_air_: air-water potential
ΔΨ _leaf−air_: the drawdown of water potential between leaf and air
ΔΨ _soil−leaf_: the drawdown of water potential between soil and leaf
V_cmax_: Maximum carboxylation rate
J_max_: maximum electron transport rate
CE: carboxylation efficiency
g_s_: stomatal conductance
g_m_: mesophyll conductance
g_tot_: total conductance
C_a_: ambient CO_2_ concentration
C_i_: intracellular CO_2_ concentration
C_C_: CO_2_ concentration of carboxylation sites inside chloroplast
L_s_: stomatal limitations imposed on the photosynthetic rate
L_m_: mesophyll limitations imposed on the photosynthetic rate
L_b_: biochemical limitations imposed on the photosynthetic rate
LMA: leaf mass area
P_n_: net photosynthetic rate
R_d_: the rate of mitochondrial respiration in the light
Γ: chloroplastic CO_2_ compensation point.
