# Supplementary material for: Physiological and Transcriptomic Analyses Revealed the Implications of Abscisic Acid in Mediating the Rate-Limiting Step for Photosynthetic Carbon Dioxide Utilisation in Response to Vapour Pressure Deficit in Solanum Lycopersicum (Tomato)
Source: Front Plant Sci. 2021 Nov 10;12:745110. doi: 10.3389/fpls.2021.745110 (PMC8631768; doi:10.3389/fpls.2021.745110)
Supplement: Supplementary file 1 [file Data_Sheet_1.PDF]

Physiological and Transcriptomic Analyses Revealed the Implications of ABA in Mediating the Rate-limiting Step for Photosynthetic CO2 Utilization in Response to the VPD in *Solanum lycopersicum* (Tomato)

Dalong Zhang[1,2,3]*, Qingjie Du[4], Po Sun[1], Jie Lou[1], Xiaotian Li[1], Qingming Li[1,2,3] and Min Wei[1,2,3]*

[1] *College of Horticultural Science and Engineering, Shandong Agricultural University, Tai'an, Shandong, China*

[2] *State Key Laboratory of Crop Biology, Tai'an, Shandong, China*

[3]*Scientific Observing and Experimental Station of Environment Controlled Agricultural Engineering in Huang-Huai-Hai Region, Ministry of Agriculture, China*

[4] *College of Horticulture, Henan Agricultural University, Zhengzhou, Henan, China*

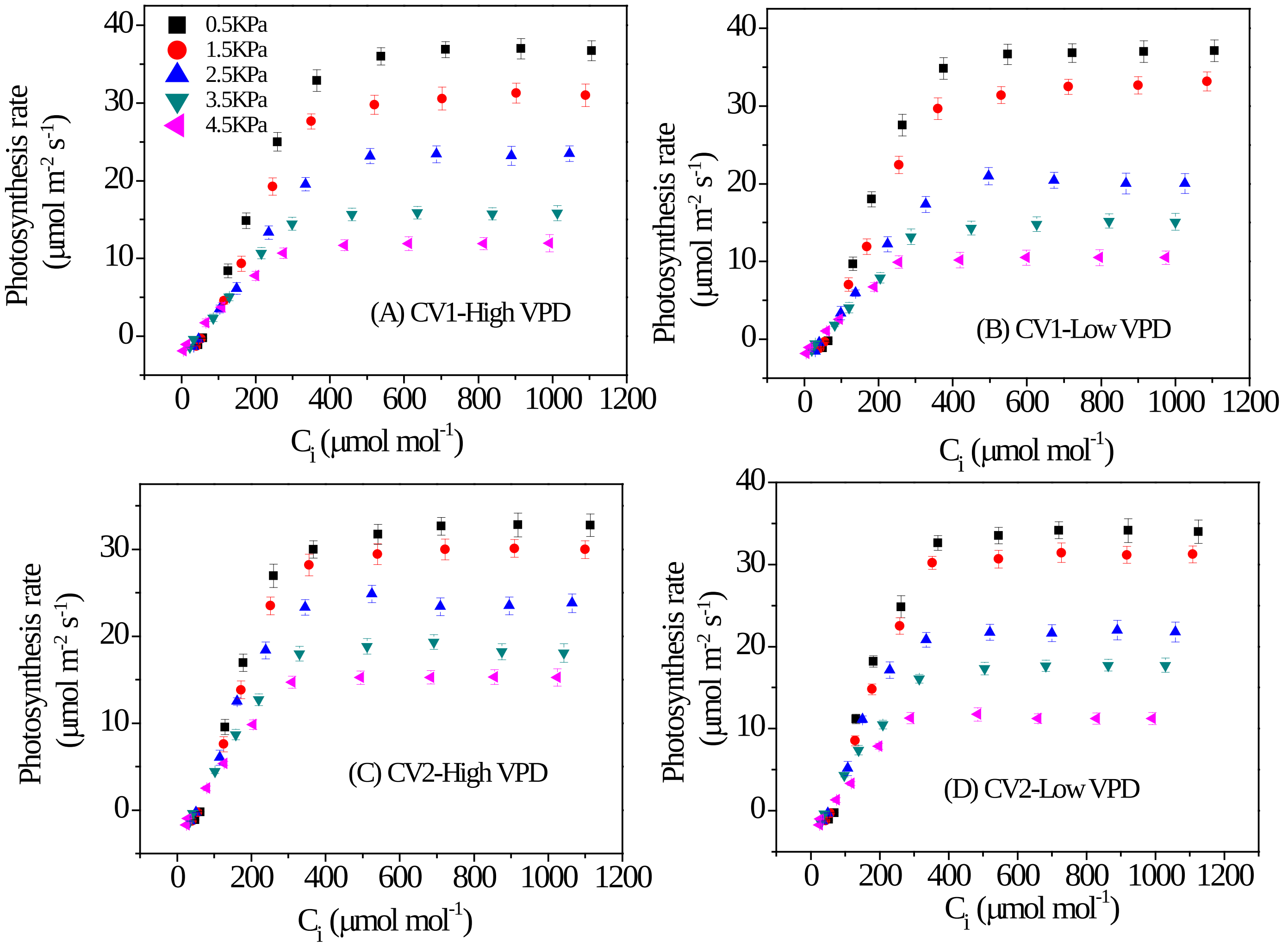

Fig. S1. Effect of VPD on photosynthetic CO_2 response curves in two tomato cultivars grown under high- and low VPD condition. Values are means ± SE (n= 4~6 replicates).

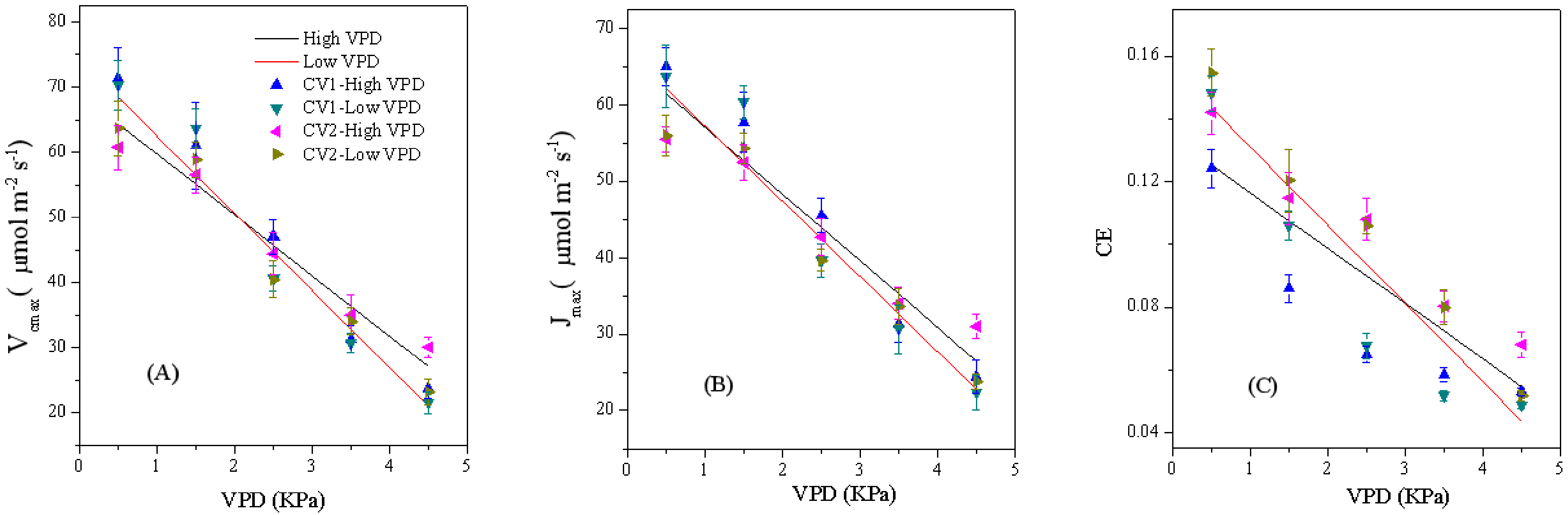

Fig. S2. Effect of VPD on photosynthetic parameters of maximum rate Rubisco carboxylation capacity (V_{cmax}), maximal rate of electron transport (J_{max}) and Carboxylation efficiency (CE). Values are means ± SE (n= 4 replicates). The regression lines shown are: (A) HVPD, V_{cmax}= -10.39VPD+72.1, R^2=0.95; LVPD, V_{cmax}= -11.82VPD+74.3, R^2=0.95. (B) HVPD, J_{max}= -8.74VPD+65.8, R^2=0.93; LVPD, J_{max}= -9.87VPD+67.1, R^2=0.93. (C) HVPD, CE= -0.0176VPD+0.134, R^2=0.74; LVPD, CE= -0.0249VPD+0.156, R^2=0.88.

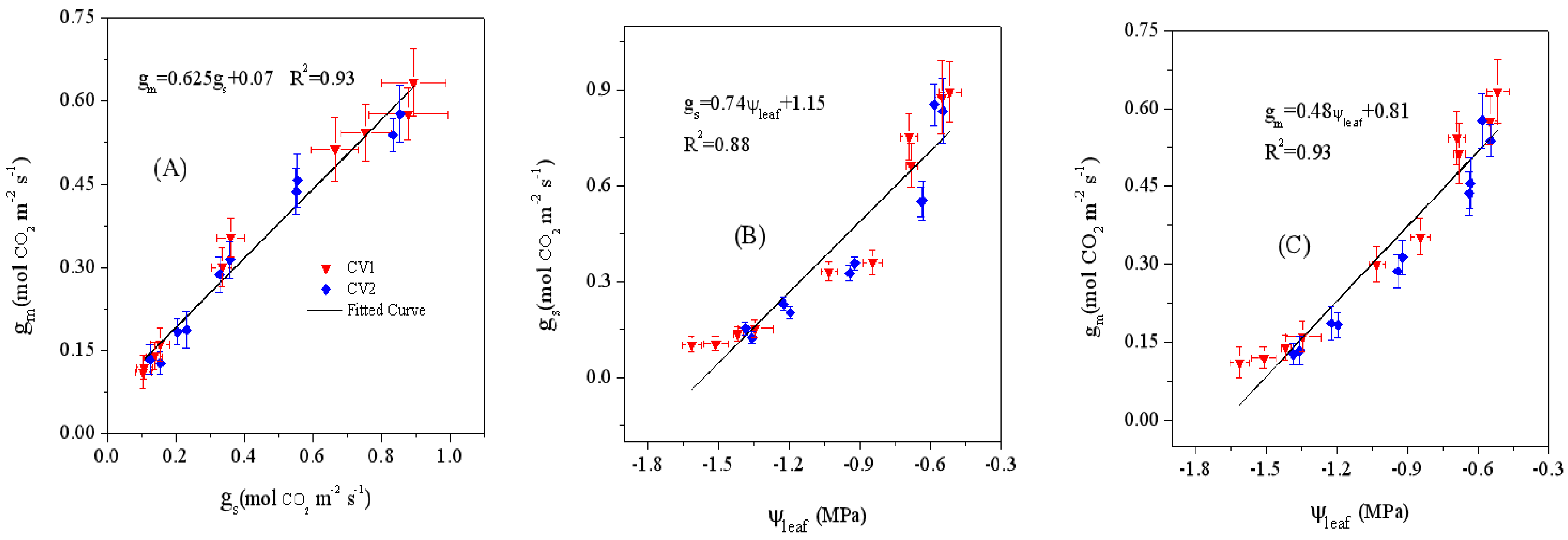

Fig. S3. Correlations between gm versus gs (A), gs versus Ψ leaf (B), and gm versus Ψ leaf (C).

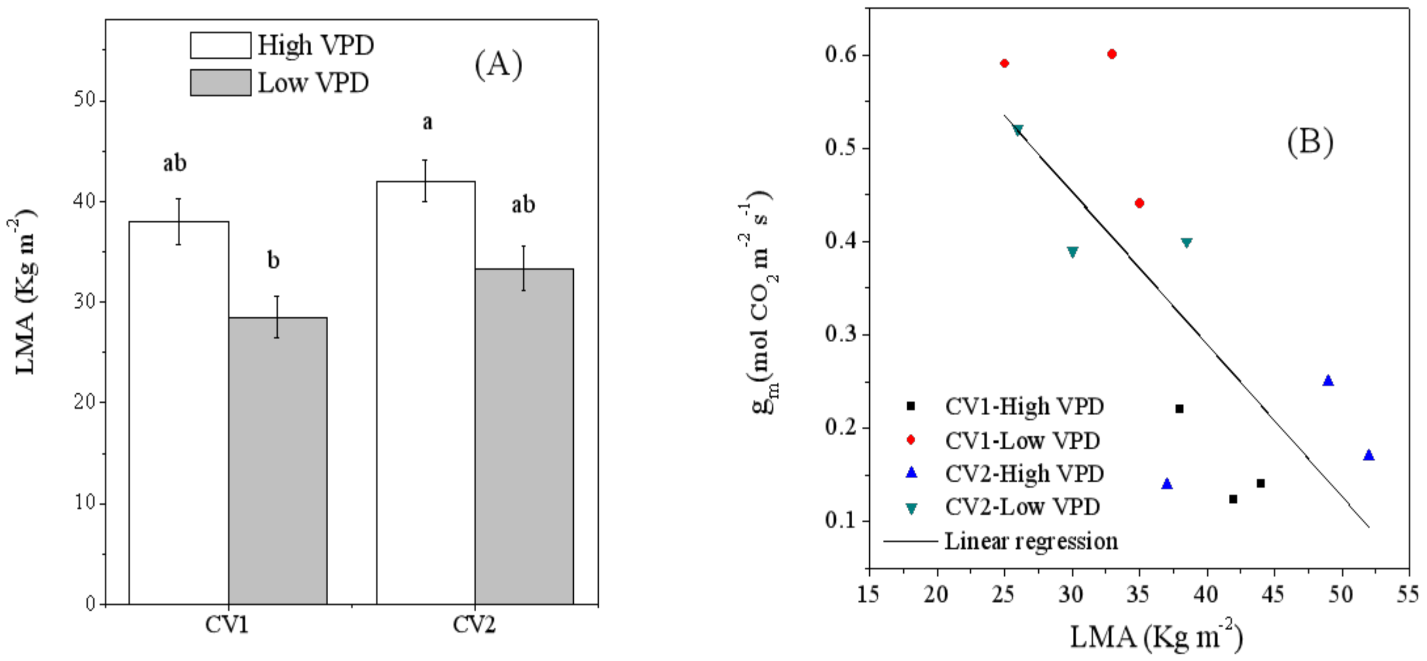

Fig. S4. Effect of VPD on leaf mass area (A; LMA) and its correction with g_m (B).

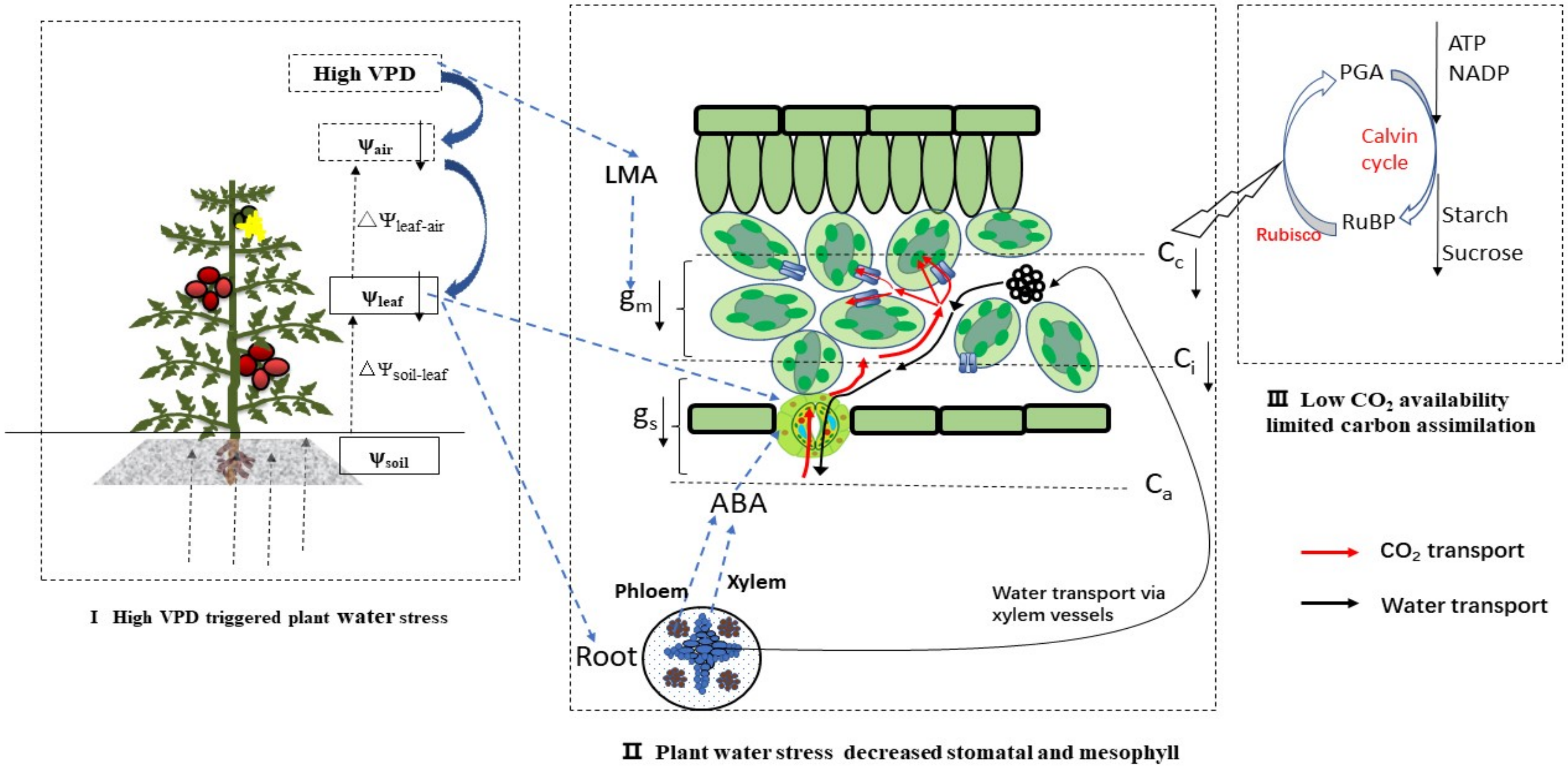

Fig.S5 A potential schematic model accounting for the increased stomatal and mesophyll limitation on photosynthesis in tomato with VPD elevation.

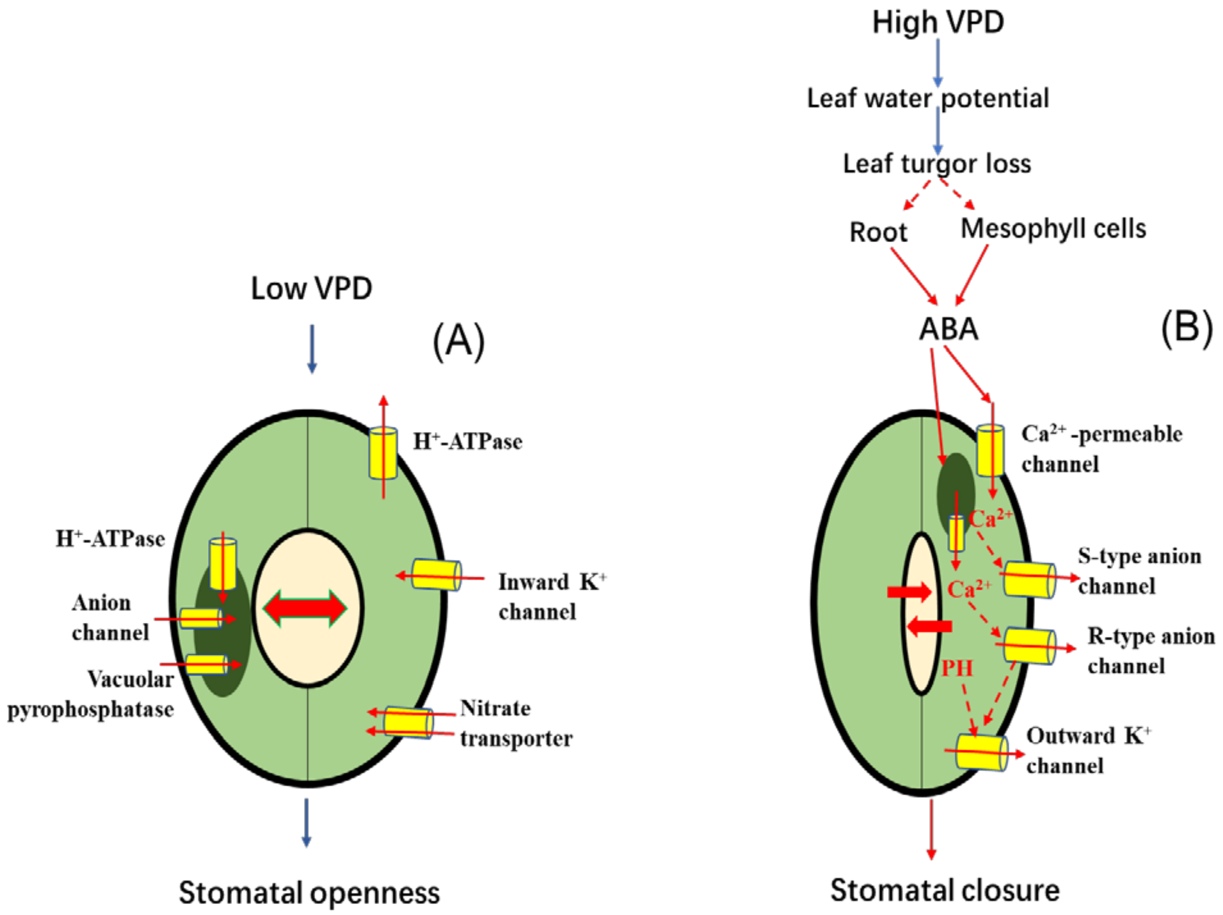

Fig.S6 Hypothetical mechanism of stomatal openness and closure response to VPD according to hydroactive- and ABA- mediated feedback model.

Supplementary Table 1. The statistical analyses of parameters of plant water status across VPD ranges

	Cultivar	Treatment	0.5 kPa	1.5 kPa	2.5 kPa	3.5 kPa	4.5 kPa
Ψ_{leaf} (- MPa)	CV1	High VPD	0.551±0.03c	0.679±0.04bc	0.846±0.04b	1.34±0.07a	1.51±0.05a
		Low VPD	0.52±0.05d	0.69±0.04d	1.03±0.04c	1.42±0.02b	1.61±0.04a
	CV2	High VPD	0.55±0.04d	0.63±0.03d	0.92±0.01c	1.22±0.01b	1.38±0.03a
		Low VPD	0.58±0.04c	0.64±0.04c	0.94±0.04b	1.19±0.03a	1.36±0.06a
$\Delta\Psi_{soil\text{-}leaf}$ (MPa)	CV1	High VPD	0.31±0.03c	0.44±0.02bc	0.61±0.04b	1.10±0.07a	1.27±0.05a
		Low VPD	0.28±0.05d	0.45±0.04d	0.79±0.04b	1.18±0.02b	1.37±0.04a
	CV2	High VPD	0.31±0.03d	0.39±0.03d	0.68±0.01c	0.98±0.01b	1.14±0.03a
		Low VPD	0.34±0.04c	0.40±0.04c	0.70±0.04b	0.95±0.03a	1.12±0.06a
$\Delta\Psi_{leaf\text{-}air}$ (MPa)	CV1	High VPD	18.95±0.03e	62.32±0.02d	123.15±0.04c	168.66±0.07b	214.49±0.05a
		Low VPD	18.98±0.05e	62.31±0.04d	122.97±0.04c	168.58±0.02b	214.39±0.04a
	CV2	High VPD	18.95±0.03e	62.37±0.03d	123.08±0.01c	168.78±0.01b	214.62±0.03a
		Low VPD	18.92±0.04e	62.36±0.04d	123.06±0.04c	168.81±0.03b	214.64±0.06a
$\frac{\Delta\Psi_{leaf-air}}{\Delta\psi_{soil-leaf}}$	CV1	High VPD	62.27±6.71c	142.80±7.52b	205.06±13.6a	154.35±11.02b	169.50±7.28ab
		Low VPD	73.270±13.9b	140.42±11.63a	156.25±6.70a	143.36±2.49a	156.41±4.93a
	CV2	High VPD	63.65±8.04b	160.66±13.03a	180.89±3.34a	171.76±2.09a	187.76±4.92a
		Low VPD	56.49±6.09b	159.95±16.43a	176.60±11.00a	177.26±5.85a	193.43±10.31a

Note: Different letters denote statistically significant differences among treatments across VPD ranges from 0.5 to 4.5kPa ($P < 0.05$). The same below.

Supplementary Table 2. The statistical analyses of photosynthetic parameters across VPD ranges

	Cultivar	Treatment	0.5 kPa	1.5 kPa	2.5 kPa	3.5 kPa	4.5 kPa
V_{cmax}	CV1	High VPD	71.3±4.81a	61.0±6.65ab	47.0±2.64bc	31.3±2.02cd	23.8±1.45d
		Low VPD	70.3±3.84a	63.7±2.96a	40.7±2.03b	30.7±1.45bc	21.5±1.73c
	CV2	High VPD	60.7±3.42a	56.5±2.77ab	44.4±3.26bc	35.0±3.05cd	30.0±1.53d
		Low VPD	63.7±4.17a	58.9±2.75a	40.5±2.78b	34.0±2.08bc	23.2±1.97c
J_{max}	CV1	High VPD	65.0±2.52a	57.7±3.92ab	45.5±2.29b	31.3±2.40c	24.4±2.12c
		Low VPD	63.7±4.10a	60.3±2.20a	39.7±2.19b	32.7±3.18bc	22.3±2.19c
	CV2	High VPD	55.4±1.72a	52.4±2.34a	42.7±2.40b	34.0±2.08bc	31.0±1.52c
		Low VPD	56.0±2.64a	54.3±2.03a	39.7±1.45b	33.7±2.33b	23.8±1.02c
CE	CV1	High VPD	0.124±0.006a	0.086±0.005b	0.065±0.003c	0.058±0.002c	0.053±0.001c
		Low VPD	0.148±0.006a	0.106±0.005b	0.068±0.004c	0.052±0.002cd	0.049±0.001d
	CV2	High VPD	0.142±0.007a	0.115±0.008ab	0.108±0.007bc	0.080±0.005cd	0.068±0.004d
		Low VPD	0.155±0.008a	0.120±0.010b	0.106±0.003bc	0.080±0.006cd	0.052±0.002d
g_s	CV1	High VPD	0.87±0.11a	0.66±0.07a	0.36±0.04b	0.15±0.03b	0.11±0.02b
		Low VPD	0.89±0.09a	0.75±0.07a	0.33±0.03b	0.14±0.02b	0.10±0.02b
	CV2	High VPD	0.83±0.10a	0.55±0.06b	0.36±0.02bc	0.23±0.02c	0.15±0.02c
		Low VPD	0.85±0.07a	0.55±0.05b	0.33±0.02c	0.20±0.02cd	0.12±0.02d
g_m	CV1	High VPD	0.58±0.05a	0.51±0.06ab	0.35±0.04b	0.16±0.03c	0.12±0.02c
		Low VPD	0.63±0.06a	0.54±0.05a	0.30±0.03b	0.14±0.02b	0.11±0.03b
	CV2	High VPD	0.54±0.03a	0.46±0.05ab	0.31±0.03bc	0.19±0.03cd	0.13±0.02d
		Low VPD	0.58±0.05a	0.44±0.04ab	0.29±0.03bc	0.18±0.02c	0.13±0.03c
g_t	CV1	High VPD	0.34±0.02a	0.29±0.03a	0.18±0.02b	0.08±0.01c	0.06±0.01c
		Low VPD	0.37±0.03a	0.31±0.01a	0.16±0.01b	0.07±0.004c	0.05±0.003c
	CV2	High VPD	0.32±0.007a	0.25±0.03b	0.17±0.01c	0.10±0.01cd	0.07±0.01d
		Low VPD	0.34±0.03a	0.24±0.002b	0.15±0.01c	0.10±0.01cd	0.06±0.01d

Supplementary Table 3. The statistical analyses of CO_2 concentration along "source-path-sink"

	Cultivar	Treatment	0.5 kPa	1.5 kPa	2.5 kPa	3.5 kPa	4.5 kPa
C_i	CV1	High VPD	359.33±7.17a	351.33±6.89a	335.33±6.49a	298.33±6.36b	276.60±4.69b
		Low VPD	362.00±6.81a	350.70±8.96a	327.00±8.74a	288.00±7.23b	255.33±6.77b
	CV2	High VPD	355.67±8.41a	348.00±8.19a	334.33±7.69ab	321.00±7.37ab	306.67±9.87b
		Low VPD	357.66±6.98a	344.67±6.44ab	335.33±8.67ab	315.00±7.21bc	290.33±6.96c
C_c	CV1	High VPD	308.33±6.06a	295.00±7.09ab	277.66±6.36b	209.33±6.49c	172.00±5.13d
		Low VPD	312.33±7.62a	295.33±8.41ab	270.33±6.57b	193.47±7.88c	155.00±6.66d
	CV2	High VPD	298.33±6.74a	285.67±8.69a	264.33±8.25a	225.33±6.69b	192.33±7.42b
		Low VPD	300.33±4.18a	272.66±5.36ab	265.57±6.83b	220.00±7.23c	200.00±6.81c
C_i/C_a	CV1	High VPD	0.89±0.02a	0.88±0.02a	0.84±0.02a	0.75±0.02b	0.69±0.01b
		Low VPD	0.91±0.02a	0.88±0.02a	0.82±0.02a	0.72±0.02b	0.64±0.02b
	CV2	High VPD	0.89±0.02a	0.87±0.02a	0.84±0.02ab	0.80±0.02ab	0.77±0.02b
		Low VPD	0.89±0.02a	0.86±0.02ab	0.84±0.02ab	0.79±0.02bc	0.73±0.02c
C_c/C_a	CV1	High VPD	0.77±0.02a	0.74±0.02ab	0.69±0.02b	0.52±0.02c	0.43±0.02d
		Low VPD	0.78±0.02a	0.74±0.02ab	0.68±0.02b	0.48±0.02c	0.39±0.02d
	CV2	High VPD	0.75±0.02a	0.71±0.02a	0.66±0.02a	0.56±0.02b	0.48±0.02b
		Low VPD	0.75±0.01a	0.68±0.01ab	0.66±0.02b	0.55±0.02c	0.50±0.02c
C_c/C_i	CV1	High VPD	0.86±0.03a	0.84±0.04a	0.83±0.05a	0.70±0.07b	0.62±0.02b
		Low VPD	0.86±0.01a	0.84±0.04a	0.82±0.01a	0.67±0.03b	0.61±0.01b
	CV2	High VPD	0.84±0.04a	0.82±0.04a	0.79±0.01a	0.70±0.03ab	0.63±0.02b
		Low VPD	0.84±0.01a	0.79±0.01ab	0.79±0.02ab	0.70±0.02b	0.69±0.04b

Supplementary Table 4. The statistical analyses of ABA and photosynthetic limitations

	Cultivar	Treatment	0.5 kPa	1.5 kPa	2.5 kPa	3.5 kPa	4.5 kPa
L_s	CV1	High VPD	15%±1%d	18%±1%cd	25%±6%bc	32%±1%ab	38%±4%a
		Low VPD	15%±1%d	16%±0.3%d	26%±1%c	33%±1%b	37%±1%a
	CV2	High VPD	15%±0.5%d	20%±1%c	24%±1%b	25%±1%b	29%±1%a
		Low VPD	15%±0.5%d	20%±0.6%c	26%±0.3%b	27%±1%b	34%±1%a
L_m	CV1	High VPD	22%±1%c	24%±1%bc	26%±1%b	30%±1%a	32%±1%a
		Low VPD	20%±1%c	23%±0.3%c	28%±1%b	32%±0.4%a	34%±1%a
	CV2	High VPD	24%±1%c	20%±1%c	29%±1%b	30%±1%b	35%±0.4%a
		Low VPD	22%±1%d	26%±1%c	28%±1%bc	30%±1%ab	32%±1%a
L_b	CV1	High VPD	63%±1%a	59%±2%ab	49%±1%bc	38%±2%cd	30%±4%d
		Low VPD	65%±1%a	61%±1%a	46%±1%b	35%±0.2%c	30%±1%d
	CV2	High VPD	62%±1%a	55%±1%b	47%±1%c	45%±0.5%c	36%±1%d
		Low VPD	63%±1%a	54%±1%b	46%±1%c	43%±1%c	34%±1%d
ABA	CV1	High VPD	361.3±8.1c	369.7±9.0c	483.7±3.94b	508.1±10.5ab	564.7±23.8a
		Low VPD	352.1±11.4c	363.1±12.9c	459.2±11.8b	514.0±14.7b	609±22.1a
	CV2	High VPD	339.3±7.5c	371.3±10.2c	445.0±9.8b	537.7±15.2a	553.3±20.6a
		Low VPD	327.3±10.3d	346.7±5.5d	441.7±11.2c	514.3±16.3b	580.3±9.6a
